# Molecular subtypes based on DNA methylation predict prognosis in lung squamous cell carcinoma

**DOI:** 10.1186/s12885-021-07807-7

**Published:** 2021-01-23

**Authors:** Xiu-Shen Li, Ke-Chao Nie, Zhi-Hua Zheng, Rui-Sheng Zhou, Yu-Sheng Huang, Zeng-Jie Ye, Fan He, Ying Tang

**Affiliations:** 1 Guangzhou University of Chinese Medicine, 12 Airport Road, Baiyun District, Guangzhou, Guangdong China; 2 Lingnan Medical Research Center of Guangzhou University of Chinese Medicine, 12 Airport Road, Baiyun District, Guangzhou, Guangdong China

**Keywords:** Lung squamous cell carcinoma, DNA methylation, Molecular subtype, TCGA, Prognosis

## Abstract

**Background:**

Due to tumor heterogeneity, the diagnosis, treatment, and prognosis of patients with lung squamous cell carcinoma (LUSC) are difficult. DNA methylation is an important regulator of gene expression, which may help the diagnosis and therapy of patients with LUSC.

**Methods:**

In this study, we collected the clinical information of LUSC patients in the Cancer Genome Atlas (TCGA) database and the relevant methylated sequences of the University of California Santa Cruz (UCSC) database to construct methylated subtypes and performed prognostic analysis.

**Results:**

Nine hundred sixty-five potential independent prognosis methylation sites were finally identified and the genes were identified. Based on consensus clustering analysis, seven subtypes were identified by using 965 CpG sites and corresponding survival curves were plotted. The prognostic analysis model was constructed according to the methylation sites’ information of the subtype with the best prognosis. Internal and external verifications were used to evaluate the prediction model.

**Conclusions:**

Models based on differences in DNA methylation levels may help to classify the molecular subtypes of LUSC patients, and provide more individualized treatment recommendations and prognostic assessments for different clinical subtypes. GNAS, FZD2, FZD10 are the core three genes that may be related to the prognosis of LUSC patients.

**Supplementary Information:**

The online version contains supplementary material available at 10.1186/s12885-021-07807-7.

## Background

Lung cancer, the most commonly diagnosed cancer (11.6%), is the leading cause of cancer death, which accounts for 18.4% of cancer deaths among men and women [1]. The incidence of lung cancer is increasing rapidly, causing a huge economic burden. Approximately 66% patients have lost the opportunity to undergo radical surgery after the diagnosis of lung cancer in China [2]. Non-small cell lung cancer (NSCLC), a heterogeneous disease, accounts for more than four-fifths of all lung cancers, and the pathological classification and clinical stage of patients are closely related to their prognosis [3]. Patients with LUSC account for more than 30% of patients with NSCLC [4].

In the past two decades, the epigenetic understanding of lung cancer has developed exponentially [5]. Epigenetic research has provided key data for the occurrence of lung cancer. DNA methylation is the presence of methyl cohorts at the CpG dinucleotides, which usually locates near the gene promoter and affects gene expressions [6]. Transcriptional silencing caused by hypermethylation of CpG islands has become a key factor in the occurrence and development of lung cancer [7]. Abnormal DNA methylation silences the expression of tumor suppressor genes (TSG) by methylation of the promoter regions [8, 9].DNA methylation markers have important value in the early diagnosis of lung cancer, predicting the treatment effect and tracking the resistance of treatment. The methylation of p16INK4a and MGMT, which can be detected in the sputum of most LUSC patients, could be used to predict the risk of lung cancer in smokers [5]. However, a large number of patients with LUSC have not been screened for abnormal methylation genes in the promoter region, and further analysis of their association with tumor classification and patient survival time. In this research, based on the TCGA and UCSC databases, we screened out multiple DNA methylation biomarkers to construct and verify the prognostic prediction model, which could be used to provide more individualized treatment recommendations and prognostic assessments for different clinical subtypes.

## Methods

### Data download and preprocessing

Downloaded RNA sequencing data which came from 504 primary LUSC samples from the TCGA databases (https://cancergenome.nih.gov/ 2020-07-08). Additional file 1 shows the clinical information of these 504 patient samples, including follow-up data. Downloaded the DNA methylation data of Illumina Infinium HumanMethylation450 and 27 BeadChip arrays, respectively.

This study only includes sample data with clinical follow-up times exceeding 30 days. β-value ranging from 0 (unmethylated) to 1 (fully methylated) represented the DNA methylation level of each site. Use the “impute” and “sva” packages in R language to eliminate the effect of batch effect. CpG sites were filtered by using the next 4 processes: (1) Remove the CpG sites with data less than 30% of samples. (2) Remove the unstable CpG sites which located on single nucleotide polymorphisms and sex chromosomes. (3) only the CpG sites in the promoter region (2 kb upstream to 0.5 kb downstream from the transcription start site) were retained. (4) Remove the CpG sites which in the Illumina Infinium HumanMethylation 450 microarray existed the polymorphic CpG and cross-reactive probes. (5) CpG sites which existed in the DNA methylation data of the downloaded Illumina Infinium HumanMethylation 27 and 450 BeadChip arrays were retained. The data from HumanMethylation 450 microarray is classified as the training cohort, and the data from 27 BeadChip is classified as the external test cohort. Additional files 2 and 3 show the methylation site profiles and clinical information of the two cohorts, respectively.

### Selecting characteristic CpG sites

CpG sites were selected by using the next 3 processes:(1) For each CpG site, TNM stage, age, gender and survival data, use methylation levels to construct a univariate Cox proportional risk regression model. (2) Introduce Sites with significantly different levels of methylation expression (*p* ≤ 0.05) from univariate Cox proportional hazards regression model into multivariate Cox proportional risk regression models constructed from TNM staging, age, gender and survival data.(3) Select the characteristic CpG sites which are significant in multivariate and univariate Cox regression analysis.

### Identification molecular subtypes related to prognosis

Based on the CpG sites with significantly different levels of methylation expression “ConsensusClusterPlus” package in R was used for consensus clustering to identify LUSC molecular subtypes. This algorithm, one of the unsupervised class discovery algorithms, defined “consensus” clustering by estimating the stability of clustering results by applying a specific clustering means to the random subsets of data. After 100 iterations, we gained the project-consensus results and cluster consensus.

The heatmap of the consensus matrix which came from the graphical output results included the consensus cumulative distribution function (CDF) plots, delta area plots and clustering results. According to the following criteria the sorts of clusters were determined: (1) The consistency within the cluster was high. (2) The coefficient of variation is relatively low. (3) The area under the CDF curve did not increase significantly. The area under the CDF curve was used to define the category number. Tend to use more categories for LUSC to get more detailed classification categories. Utilize the “pheatmap” R package to get heatmap which correspond to consensus clustering. Use a color gradient to indicate the consensus value from 0 (white) to 1 (dark blue); sort out the matrix so that the items which belongs to the same cluster can be put together. In this arrangement, the perfect consensus matrix will show a heatmap, which is displayed by the diagonal blue blocks on a white background.

### Analyses of survival and clinical characteristics

Use Kaplan–Meier plots to demonstrate overall survival among LUSC subtypes which was determined by DNA methylation profiles. Utilize the log-rank test to assess the significance of differences among the subtypes. Use the “survival” R package to execute survival analyses. The chi-squared test was used to analysis the connection between DNA methylation clustering and both biological and clinical characteristics. All tests used two-sided, and only *p* < 0.05 was thought statistically significant.

### GO and KEGG enrichment analysis

The “clusterProfiler” and “ggplot2” R package combined with Gene Ontology (GO) and Kyoto Encyclopedia of Genes and Genomes (KEGG) database were applied to process gene enrichment analysis of the biological process, cell component, molecular function and biological pathways.

### Construction and testing of the prognostic prediction model

Based on the prognostic information of patients and methylation profiles of 26 CpG sites, Use the “survival” R package to construct and verify the Cox proportional hazards model. The formula of the model is: risk score = cg01775414*7.35-cg03522063*1.71-cg03954587*0.65 + cg05158615*0.49-cg06918467*0.55-cg07850604*0.08-cg09339527*0.46-cg10332700*0.07-cg11814446*1.64 + cg11846236*2.60 + cg13739417*2.52-cg14776962*0.38-cg15001381*6.07-cg17619823*0.12 + cg19255783*0.10 + cg20855565*0.05-cg21331821*0.33 + cg21663122*1.51-cg23054883*3.52 + cg24402880*0.42 + cg24892510*0.90-cg25228126*0.08 + cg25533774*0.56-cg25856383*0.93-cg25983380*0.30-cg27654142*1.88. Internal verification randomly divided the data from the 450 k BeadChip into an internal training cohort and an internal testing cohort for verification, while external verification used all data of the 450 k BeadChip as the external training cohort and all data of the 27 k BeadChip as the external testing cohort.

We probed the genetic alterations connected with the 24 genes corresponding to 26 prognostic-related methylation sites, and the correlation between messenger RNA (mRNA) and DNA methylation by utilizing the cBioPortal tool. The clinical characteristics and prognosis model of the patient were used as different influencing factors, and the prognostic information of the patient was analyzed by univariate and multivariate Cox analyses, and the corresponding ROC curve is drawn.

## Results

### Select potential methylation sites associated with the prognosis of patients

After preprocess the downloaded patients data according to the description in Materials and Methods, we identified 21,122 methylation sites. Then, the patients were divided into two cohorts, namely the training cohort and the test cohort, and detailed patient data is shown in the Additional files 2, 3. With *p* < 0.05 as the screening condition, the univariate Cox regression analysis was used to select the CpG sites, which could be used to serve as potential DNA methylation biomarkers for overall survival of patients with LUSC. Finally, obtain 1160 related CpG sites (Additional file 4). The T, N, M, stage and age as covariates Multivariate Cox regression analysis was performed on 1160 methylation sites, and 965 independent CpG sites, which were considered potential prognostic methylation sites were identified (Additional file 5).

### Consensus clustering to identify different subtypes of DNA methylation prognosis and prognostic analysis among subtypes

**Fig. 1:**
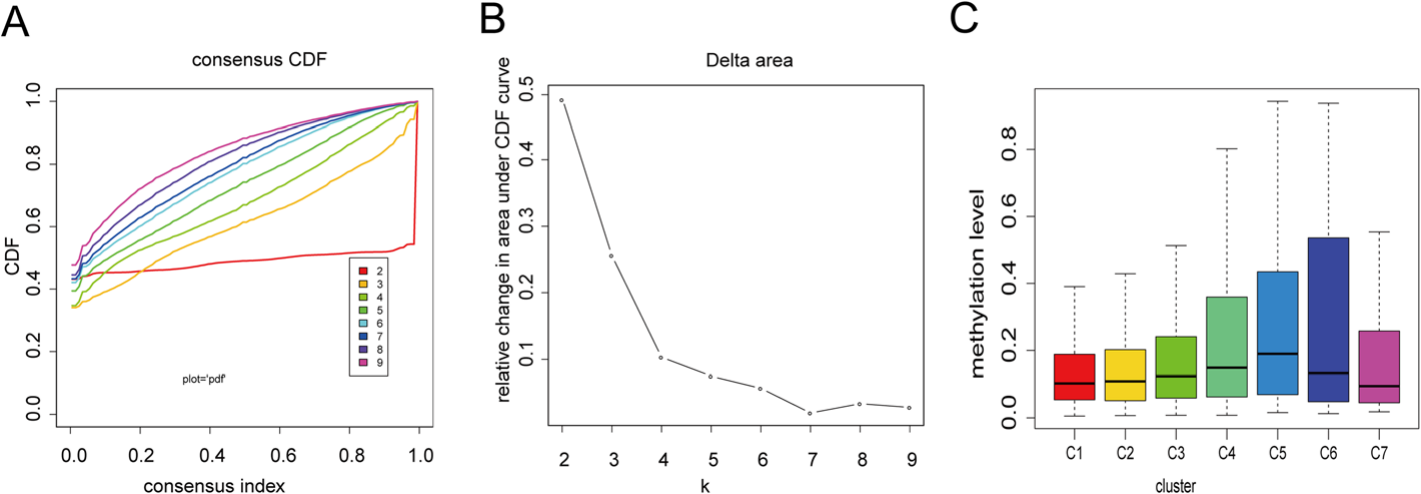
Standard for choosing amount of subtypes. **a** Consensus among subtypes for each cluster number k. **b** The Delta area curve used for consensus clustering represents under the CDF curve the relative change in the area for each cluster number k compared to k-1. The category number k is indicated by the horizontal axis coordinate, and the relative change of the area under the CDF curve is indicated by the vertical axis coordinate. **c** Box plot of CpG methylation levels of the 7 Clusters. (All figuress were generated by “R” (3.6.2) software and Adobe Illustrator (23.0.2) software)

**Fig. 2:**
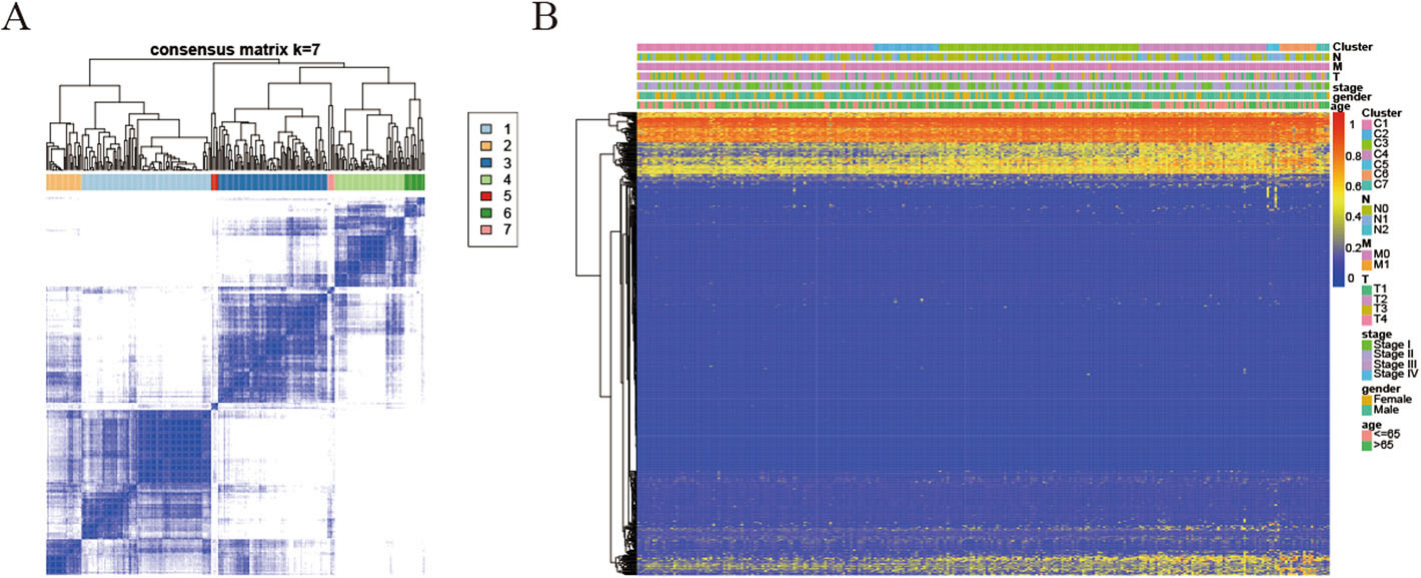
Consensus matrix and corresponding heat map of DNA methylation subtypes. **a** Color-coded heatmap which corresponds to the consensus matrix(k = 7) gained by processing consensus clustering. Use a color gradient to indicate the consensus value from 0 (white) to 1 (dark blue). **b** A heatmap which corresponds to the Fig. 2**a** with the annotation of the DNA methylation subtypes, stage,age and TNM category. (All figuress were generated by “R” (3.6.2) software and Adobe Illustrator (23.0.2) software)

The consensus clustering of 965 potential prognostic methylation sites were utilize to identify different subtypes of LUSC for prognostic purposes. The amounts of clusters were decided by using the next 2 standards: (1) The consistency within the cluster is high. (2) The area under the CDF curve is not increase significantly. Based on the category number, the average clustering consensus was calculated. As shown in Figs. 1a and b, after six categories the area under the curve (AUC) of CDF started to stabilize. We chose a larger number of clusters to increase the prognostic value of LUSC subtypes. Next, we also utilized the consensus matrix to decide the optimal amount of subtypes. As shown in Fig. 2a, the consensus matrix, which showed a well-defined block structure represented the consensus of 7. DNA methylation subtypes, stage, age and TNM category are displayed as annotations in Fig. 2b which corresponds to the dendrogram of the Fig. 2a.

**Fig. 3:**
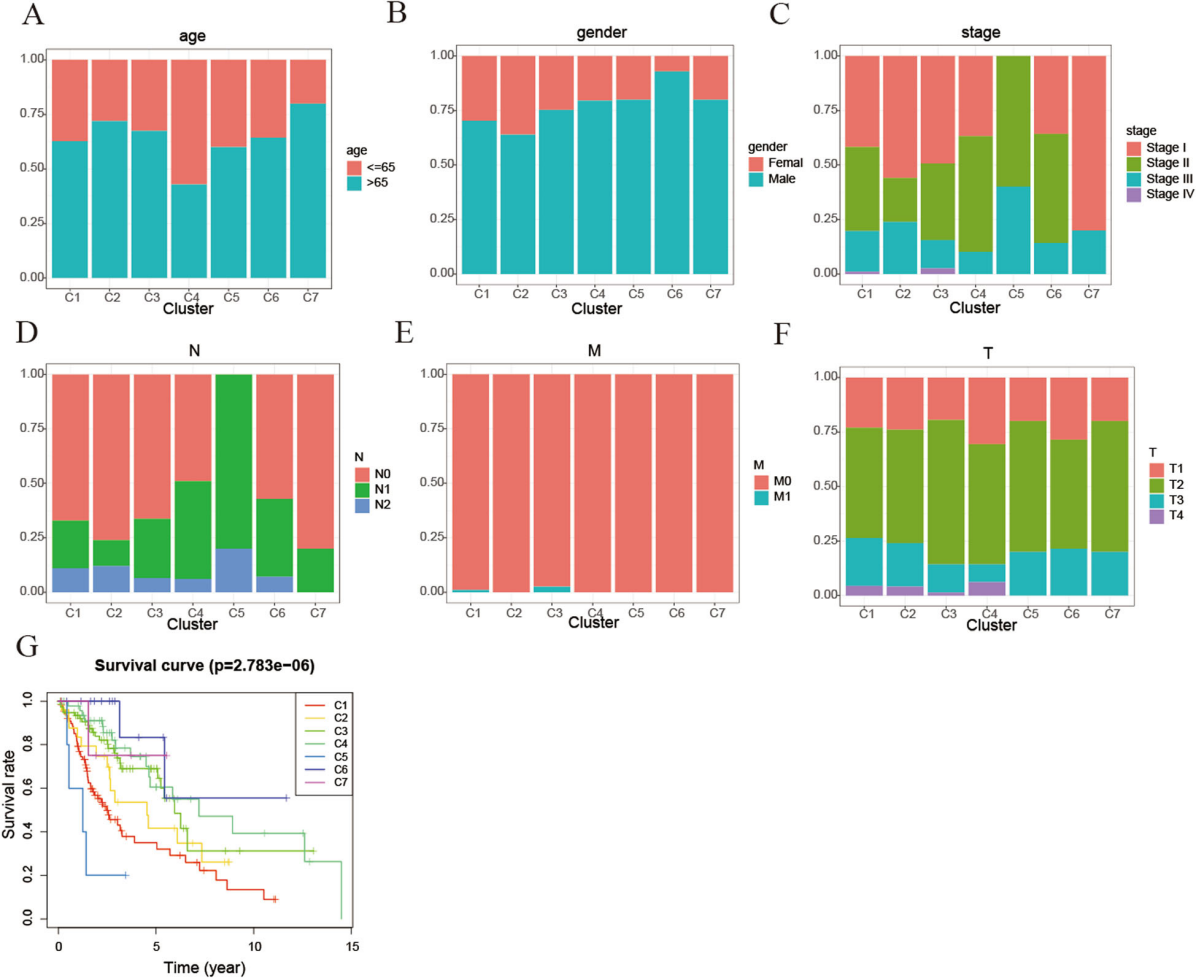
Comparison of age, gender, stage, TNM stage and prognosis among the DNA methylation subtypes. **a**, **b**, **c**, **d**, **e**, and **f**represent age, gender, stage score, topography score, lymphocyte infiltration and metastasis distributions for every DNA methylation cluster in the training set. The DNA methylation subtypes are represented by the coordinates of the horizontal axis. **g** Survival curves for every DNA methylation cluster in the training group. The survival time (years) is represented by the horizontal axis, and the survival probability is represented by the vertical axis. Use the log-rank test to evaluate the statistical significance of differences among clusters. (All figures were generated by “R” (3.6.2) software and Adobe Illustrator (23.0.2) software)

The Kaplan-Meier survival analysis showed significant differences in prognosis between the 7 subtypes (*p* < 0.001). The proportions were shown in Fig. 3a-f. The correlation tendencies among the 7 clusters were shown in the figure: (1) Cluster 4 had the smallest proportion of patients over 65 years of age, while Cluster 7 had the highest proportion of patients over 65 years of age. (2) The proportion of female patients in cluster 2 was the largest, while the proportion of female patients in cluster 6 is the smallest. (3) Cluster 7 has the largest proportion of patients in stage I, and Cluster 5 had the smallest proportion of patients in stage I. (4) Cluster 4 had the largest proportion of patients with T1. (5) Cluster 7 had the largest proportion of patients with N0, and Cluster 5 had the smallest proportion of patients with N0. (6) Almost all patients did not have distant metastasis. These tendencies revealed that each clinical parameter had a different ratio among 7 clusters. As shown in Fig. 3g, cluster 5 has the worst prognosis, while cluster 7 has the best prognosis. Then base on the clinical information of age, gender, stage score, topography score, lymphocyte infiltration and metastasis, we analyzed intra-subtype ratio for the 7 subtypes.

### Recognize different features according to DNA methylation clustering and selecting the cluster-specific methylation sites

**Fig. 4:**
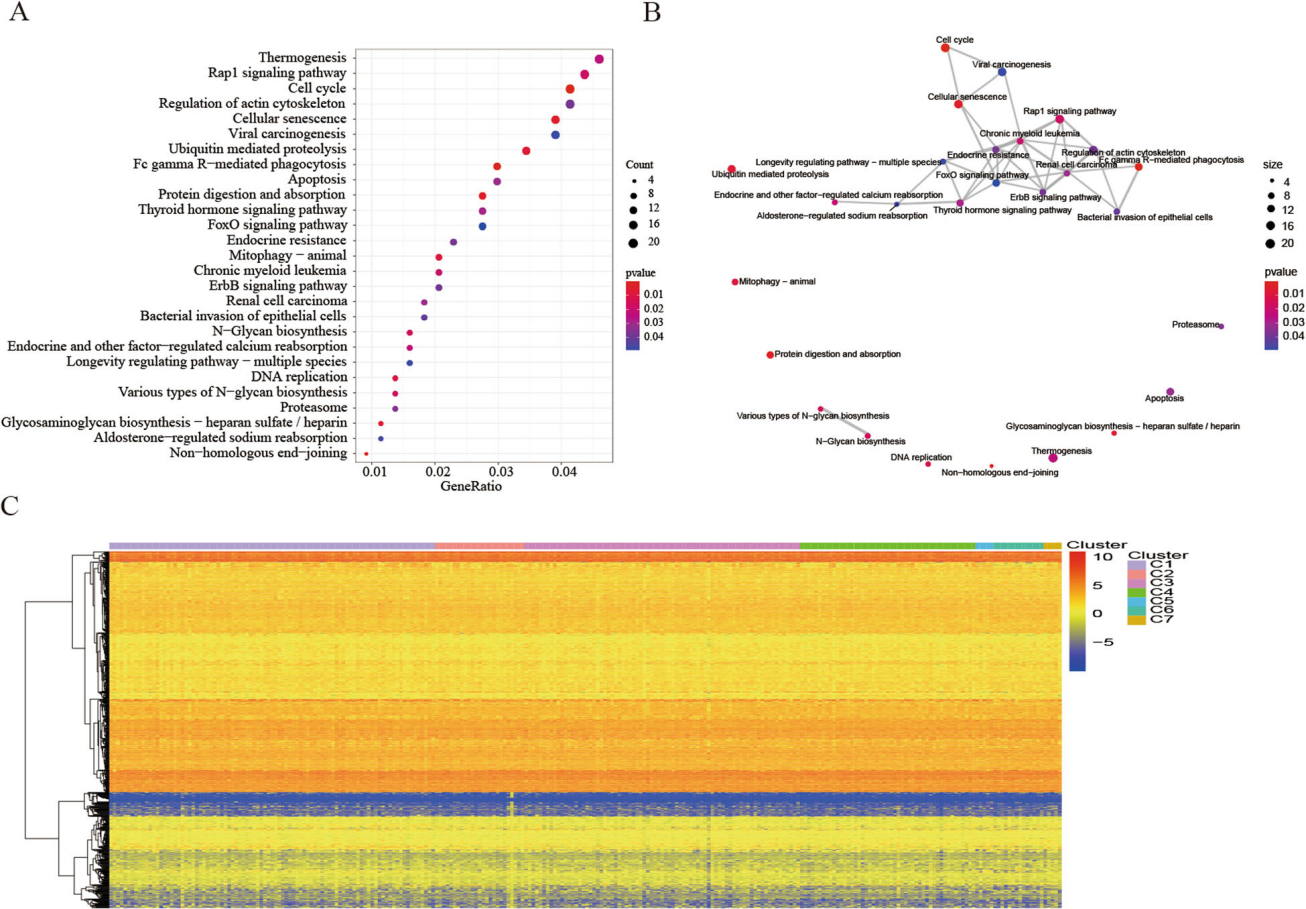
Gene annotations of 965 methylated sites. **a** KEGG pathway enrichment analysis of annotated1037 genes for the 965 CpG sites. **b** Crosstalk analysis of the results from KEGG pathways enrichment analysis using “Enrichment” R package. **c** Cluster analysis heat map for annotated genes associated with the 966 CpG sites. (All figuress were generated by “R” (3.6.2) software and Adobe Illustrator (23.0.2) software)

Though the above-mentioned genome annotations for the 965 CpG sites, we identified 1037 corresponding genes altogether. Next these 1037 genes were identified 27 significant enrichment pathways (*P* < 0.05) which were displayed in Fig. 4 and Additional file 6 by processing KEGG pathway enrichment analysis. Cell cycle, Fc gamma R-mediated phagocytosis, Non − homologous end−joining, Protein digestion and absorption, Cellular senescence was the top five pathways with significant differences. Using “enrichplot” R package to analysis the crosstalk of pathways identified by KEGG pathway enrichment analysis (Fig. 4b). Renal cell carcinoma, chronic myelogenous leukemia, and FoxO signaling pathway were the three most connected pathways with other signaling pathways.

**Fig. 5:**
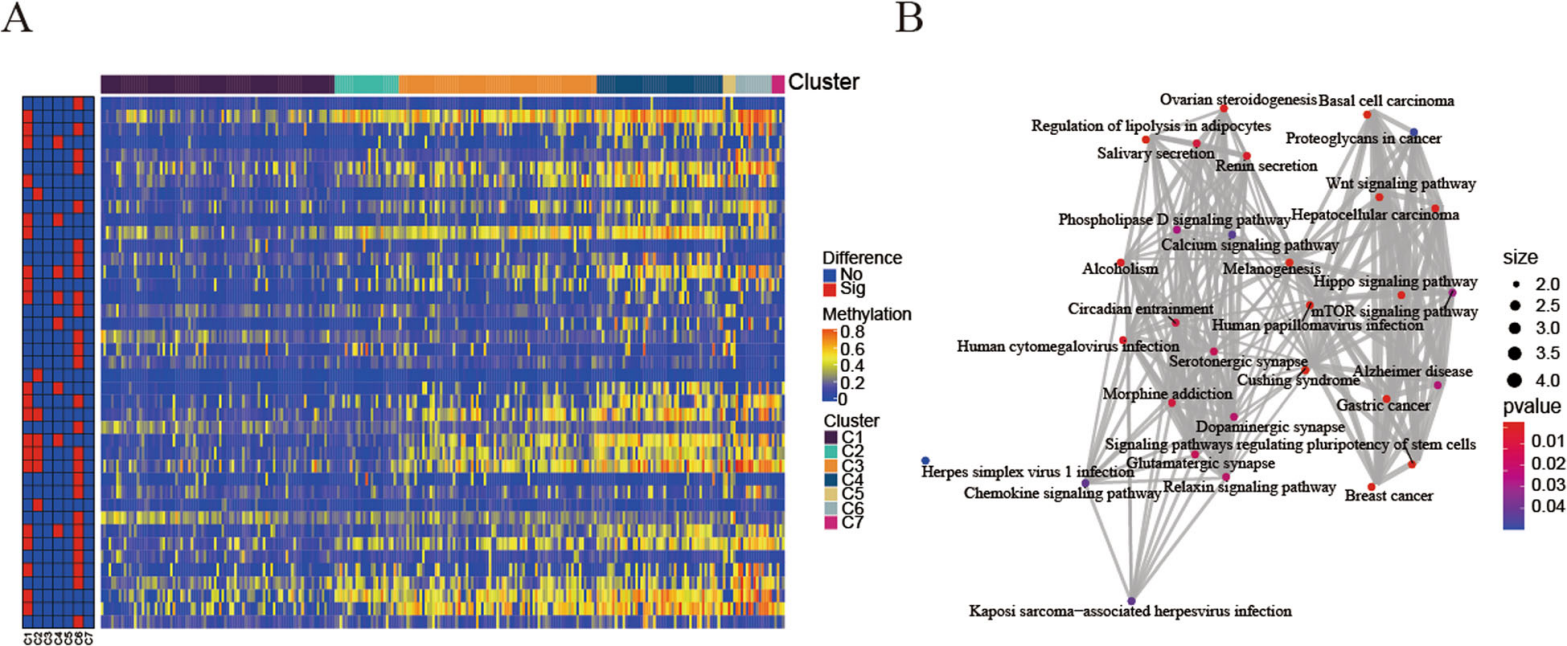
Specific hypo/hyper-methylation CpG sites for every DNA methylation cluster. **a** Specific CpG sites are displayed for every DNA methylation prognosis subtype. Hypo- and hypermethylation CpG sites are represented by red and blue bars. **b** Crosstalk analysis of the results from KEGG pathways enrichment analysis using “Enrichment” R package. (All figuress were generated by “R” (3.6.2) software and Adobe Illustrator (23.0.2) software)

Then, we selected the cluster-special methylation sites by including the CPG methylation sites as features of the clusters. Nine hundred sixty-five genes in 266 external training data set samples were available. The gene expression heat map is shown in Fig. 4c, and the original data is shown in Additional file 7.As description of Materials and Methods, analyze the differences between the 7 clusters at each methylation sites (Additional file 8). Forty-one cluster-special methylation sites identified was displayed in Additional file 9. Enrichment analysis of KEGG signaling pathway was performed to analysis genes corresponding to 41 cluster-special methylation sites (Fig. 5b). The enrichment analysis of KEGG signaling pathways resulted in 30 related signaling pathways. Analysis of the genes that make up the pathway revealed that the genes GNAS, FZD2, FZD10, GNG4, and AXIN1 participated in the most relevant pathways. Genome annotations of the 41 specific sites were used to identify their corresponding genes (Additional file 10). The analysis of the 10 signaling pathways with the smallest *p*-values revealed that these 46 genes were related to diseases such as Basal cell carcinoma, Breast cancer, Gastric cancer, etc., and pathway such as Signaling pathways regulating pluripotency of stem cells, Hippo signaling pathway, Wnt signaling pathway.

The 30 signal pathways obtained by enrichment analysis were only enriched in Clusters1, 2, 4, and 6 (Additional file 11). Cluster 7 has the lowest methylation level among all clusters, while cluster 5 has the highest methylation level (Fig. 1c).

### Construction and evaluation of LUSC prognostic model

**Fig. 6:**
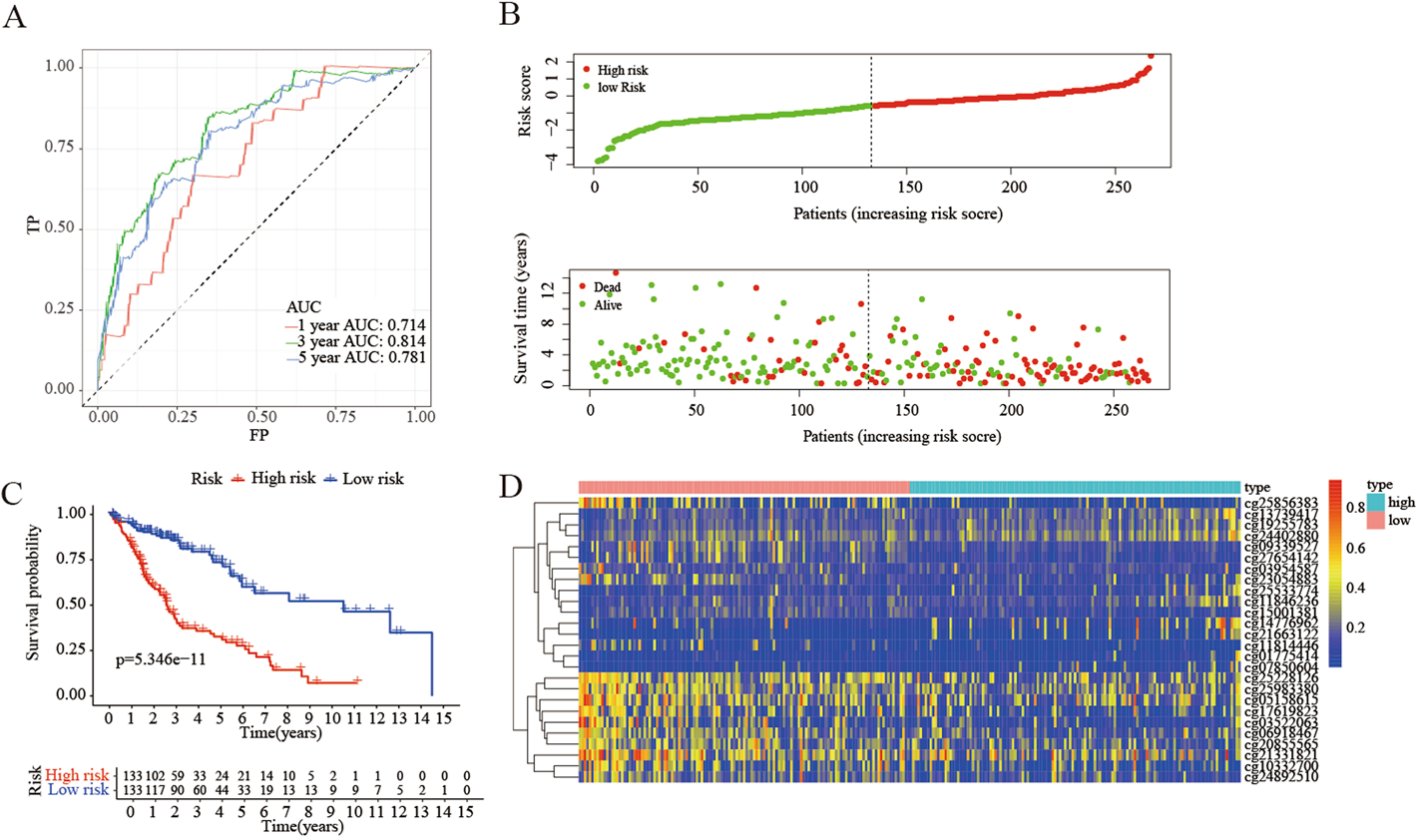
Construction of the model for forecasting prognosis of LUSC patients in the external train group. **a** The ROC curve of the prognostic indicators of 1 year, 3 years and 5 years. **b** The horizontal axis represents patient samples, and the vertical axis represents the risk score (upper) and the overall survival (lower). **c** Prognostic difference analysis between high-risk group and low-risk group. **d** The Heat map showing the expression levels of 26 methylation sites which were used to build the prognostic model in the high-risk and low-risk groups in external train group. (All figuress were generated by “R” (3.6.2) software and Adobe Illustrator (23.0.2) software)

Cluster 6 with 26 specific methylation sites was selected as the seed cluster, because it has a good prognosis and the largest number of specific methylation sites among clusters. For all samples in the Training cohort, the methylation levels of these 26 specific sites were obtained. Next, we built a Multistate Cox risk regression model, and used this model to calculate the risk value of each patient. Utilizing the “survival” and “survminer” R package to plot survival curves, the results showed that there were significant differences between the two cohorts (Fig. 6). Specifically, the prognosis of the high-risk cohort was poor, indicating that these specific methylation sites might be a sign of prognosis. The ROC analysis was performed using the risk score computed for each training cohort sample, and the results are shown in Fig. 6a.The AUC as 0.714, indicated that the model worked well. Then sort the samples by risk score and find that as the score increases, the risk of death is higher (Fig. 6b). According to the cut-off risk score of − 0.70491, the patients in the training cohort were divided into high-risk cohort and low-risk cohort evenly, and the heat map was used to show the methylation level of 26 special sites in the training cohort (Fig. 6d).

**Fig. 7:**
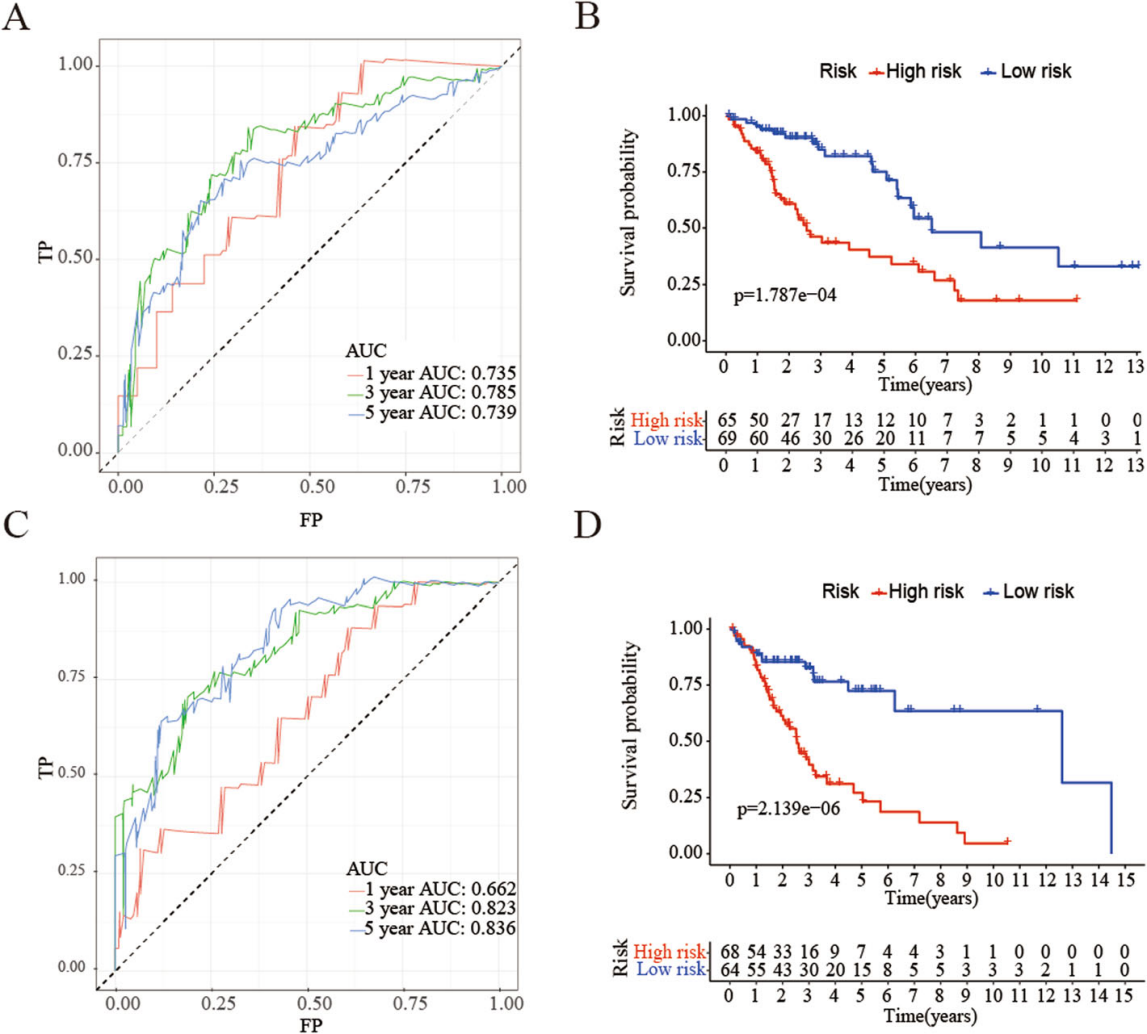
Construction and internal verification of the model for forecasting prognosis of LUSC patients. **a** ROC curves of prognostic predictors in the internal training group. **b** Prognostic difference analysis between high-risk group and low-risk group in the internal training group. **c** ROC curves of prognostic predictors in the internal testing group. **d** Prognostic difference analysis between high-risk group and low-risk group in the internal testing group. (All figuress were generated by “R” (3.6.2) software and Adobe Illustrator (23.0.2) software)

Then, we used the caret R package to divide the patients in the external training cohort into two cohorts, namely the internal training cohort and the internal test cohort. We calculated the patient’s risk value, and used the R language to draw the survival curve and ROC curve of the two cohorts of patients. As shown in Fig. 7, the prognosis of patients in the high and low risk cohorts is statistically significant, and the *p* value was less than 0.001. The AUC is 0.735 and 0.662, respectively, which in consistent with the results of the external training cohort.

**Fig. 8:**
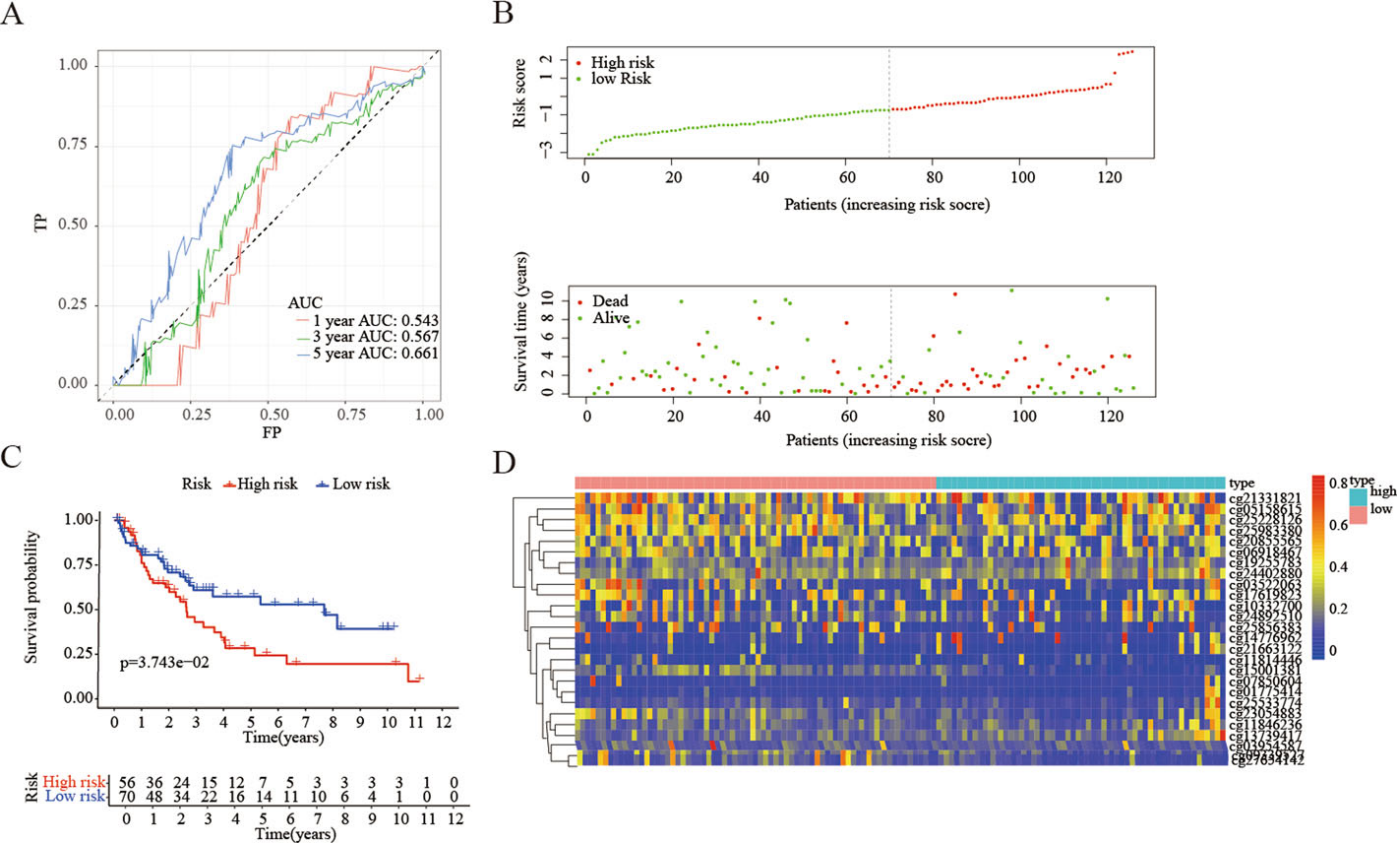
Construction of the model for forecasting prognosis of LUSC patients in the external test group. **a** The ROC curve of the prognostic indicators of 1 year, 3 years and 5 years. **b** The horizontal axis represents patient samples, and the vertical axis represents the risk score (upper) and the overall survival (lower). **c** Prognostic difference analysis between high-risk group and low-risk group. **d** The Heat map showing the expression levels of 26 methylation sites which were used to build the prognostic model in the high-risk and low-risk groups in external test group. (All figuress were generated by “R” (3.6.2) software and Adobe Illustrator (23.0.2) software)

Finally, the data of the patients in the testing cohort were collated to obtain the methylation levels of the patients at 26 special sites (Fig. 8d), and the obtained prognostic model was applied to the patients in the external test cohort which came from the 27 BeadChip to calculate their risk scores. Using − 0.70491 as the critical value, it was divided into high-risk cohort and low-risk cohort (Fig. 8b). The prognosis of the low-risk cohort was significantly better than that of the advanced cohort (Fig. 8c), and there was a significant difference between the two cohorts (*p* = 0.03743), Consistent with the results of the training cohort, it was proved that this model could be used to predict the prognosis of patients.

**Fig. 9:**
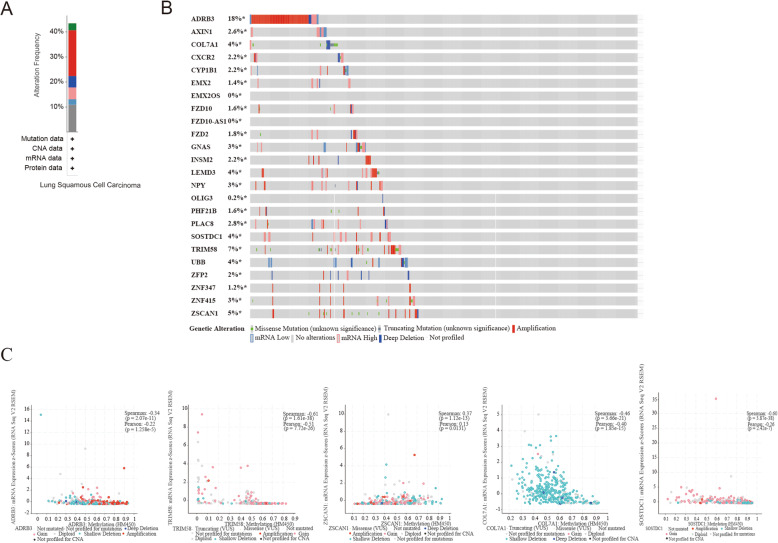
The genetic alterations associated with 24 genes and the correlation between mRNA and DNA methylation. **a** The spliced bar graph summarizes and displays the genetic variation of 24 genes in 43% of the sequenced cases/patients (511 in total). **b** The specific genetic variation of 24 genes in the TCGA lung cancer dataset. **c** The correlation between DNA methylation and mRNA of top 5 genes in the TCGA lung cancer dataset. (All figuress were generated by “R” (3.6.2) software and Adobe Illustrator (23.0.2) software)

The alteration information of the 24 genes was showed in Fig. 9. We found that the 24 genes were altered in 219 (43%) of the 511 sequenced cases/patients (511 total). The ADRB3 was altered most often (18%), including deep deletion, amplification, mRNA high, etc. The correlation between mRNA and DNA methylation of the 5 genes with highest degree of genetic alterations in the TCGA LUSC patients was demonstrated in Fig. 9c. We found that the correlation was most negative, indicating that methylation regulated the mRNA expression of these genes (except for EMX2, LEMD3, ZFP2, ZSCAN1). The results illustrated that the DNA methylation played an significant role in the expression of these genes.

**Fig. 10:**
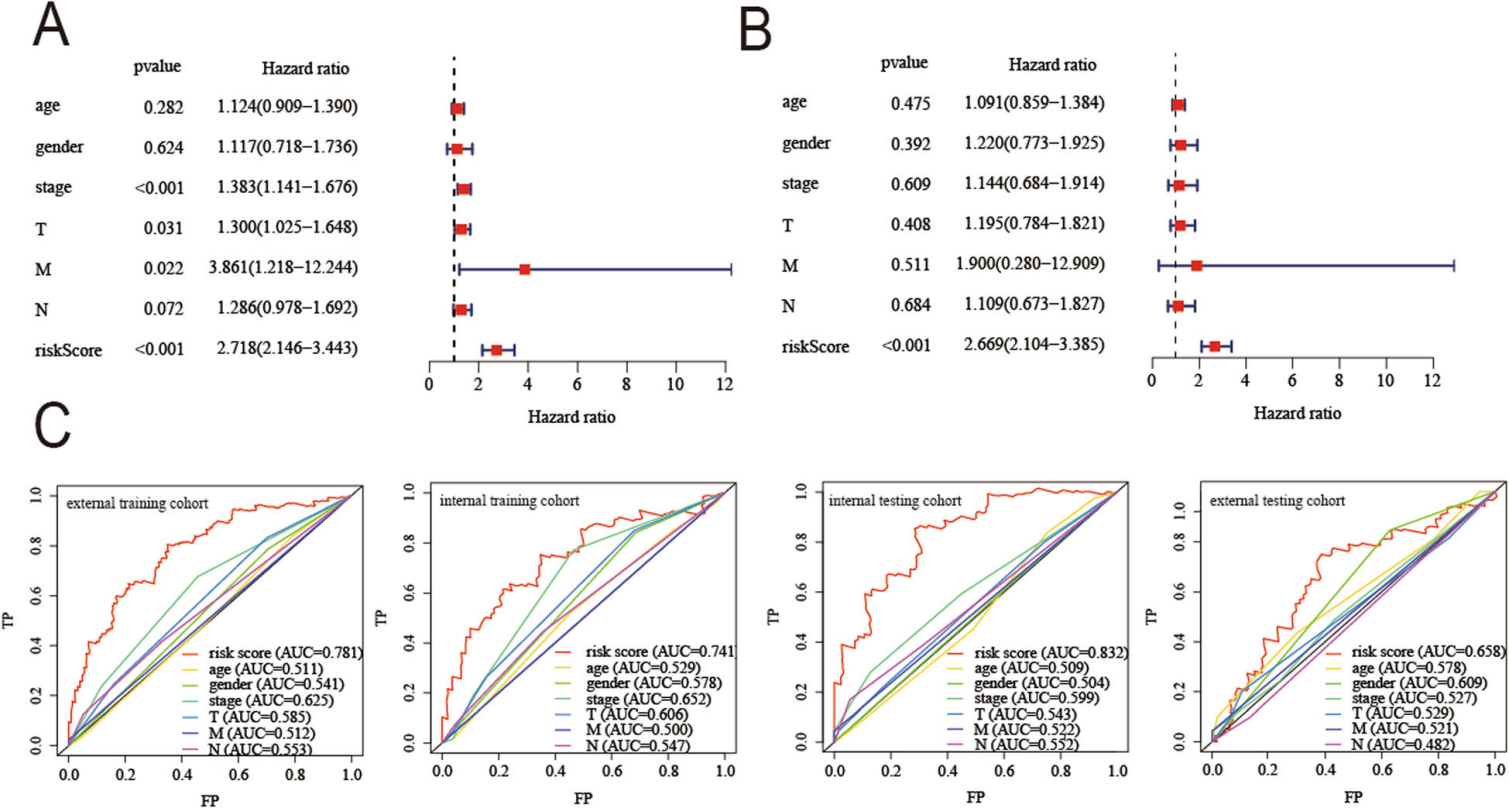
Independent predictive power of the prognosis model for LUSC. **a** Univariate Cox analysis. **b** Multivariate Cox analysis. **c** AUC in ROC analysis for prognosis model and clinical characteristics in the signature at the 5-year survival time in four cohorts. (All figuress were generated by “R” (3.6.2) software and Adobe Illustrator (23.0.2) software)

The clinical characteristics and prognosis model of the patient were used as different influencing factors, and the prognostic information of the patient was analyzed by univariate and multivariate Cox analyses, and the corresponding ROC curve is drawn. The results of the univariate Cox analysis found that the predictive effect of the prognostic model and patient stage was higher than other clinical characteristics (Fig. 10a). However the results of multivariate Cox analyses showed that only the prognostic model can independently evaluate the prognosis of the patient (Fig. 10b).

## Discussion

The study processed data of patients with LUSC which download from the TCGA database and UCSC database. Through univariate Cox regression analysis and multivariate Cox regression analysis, 965 potential prognostic methylation sites were screened out. According to the selected prognostic methylation sites, the consensus clustering method was used to identify 7 different subtypes of LUSC. The subtype with the best prognosis was selected to construct a multivariate Cox risk regression model which was used to calculate the prognostic risk value of each patient with LUSC. The survival curve found that the higher the risk value, the worse the prognosis (Fig. 6b). Internal verification and external verification found that the prognostic model has good predictive performance.

The incidence of LUSC is high, and its five-year survival rate is less than 15% [10]. Therefore, in order to increase the survival time of patients, it is urgent to integrate the clinical and related detection information of LUSC patients to identify new early diagnosis biomarkers, find new therapeutic targets, and predict the prognosis of patients. The rapid development of high-throughput sequencing technology has provided valuable data for studying the mechanism of cancer. Cancer is associated with genetics and epigenetics [11]. In all DNA-based biological processes such as transcription, modification, and replication, epigenetic modification conveys information that can play a crucial regulatory role [12]. Epigenetic changes affect the entire process of tumorigenesis and development by affecting genomic stability and gene expression [13]. Epigenetic changes occur in the early stages of tumor development and can be adjusted by external factors, such as drugs, diet, etc., so the individual’s epigenetic analysis may provide valuable information for reducing their risk of cancer [14–16]. DNA methylation, microRNA (miRNA), nucleosome remodeling and histone modification are the main mechanisms of epigenetics [17]. These four mechanisms have been proven to be associated with many diseases including tumors [18].

As an important part of epigenetics, DNA methylation has long become a research hotspot. It seems clear that the silencing expression of TSG caused by methylation may be the origin of important events in tumorigenesis [19]. There is increasing evidence that DNA methylation is associated with lung cancer [20, 21]. Studies have shown that the incidence of H-cadherin methylation in patients with NSCLC is significantly related to tumor stage [22]. The methylation levels of VAX1, CH25H, ADCYAP1 and IRX1 genes are related to the prognosis of LUSC patients [10]. The prognosis model of LUSC constructed in this study uses 26 related methylation sites, corresponding to 24 related genes. GNAS, FZD2, FZD10 are the core three genes that may be related to the prognosis of LUSC patients. GNAS mutations have been found in pancreas, colon and lung tumors, and in up to two-thirds of intraductal papillary mucinous tumors (IPMNs) [23–27]. According to the results of in vitro and in vivo experiments, Fzd2 is an oncogene, and overexpression of Fzd2 and signaling through the non-canonical Wnt pathway can promote the development of advanced metastatic cancer [28]. FZD10, a receptor for the Wnt pathway, is associated with the activation of Wnt signaling in colorectal cancer, gastric cancer, and synovial sarcoma, and is expressed at high levels in these cancers [29–31].

In summary, we constructed a prognostic model based on the methylation data of patients with lung squamous cell carcinoma. Further analysis of the ROC curve multi-year survival rate curve shows that as the survival time increases, the prediction results of the model become more accurate. The constructed prediction model integrates independent prognostic methylation sites, which can be used to identify new tumor markers, guide the clinical treatment of patients, and evaluate the prognosis of patients. This model can provide more individualized treatment recommendations and prognostic evaluations by classifying the molecular subtypes of LUSC patients.

## Conclutions

In this study, we divided patients into seven molecular subtypes based on their methylation expression levels. Based on the methylation sites of the subcohort with the best prognosis, a model that can be used to evaluate the prognosis of LUSC patients was constructed. After external and internal verification, it is found that with the extension of survival time, its prediction effect shows an upward trend. Our results indicate that DNA methylation plays an important role in the occurrence and development of LUSC and may be proposed as a diagnostic biomarker.

## Supplementary Information

**Additional file 1.** The clinical information and follow-up data of 504 patients

**Additional file 2.** The clinical information of external training dataset

**Additional file 3.** The clinical information of external testing dataset

**Additional file 4.** Univariate Cox regression analysis of the training dataset (1159)

**Additional file 5.** Multivariate Cox regression analysis of the 965 methylation sites (965)

**Additional file 6.** Functional enrichment analysis and the identified 27 enriched pathways

**Additional file 7.** The available expression profile of 965 sites in 266 training set samples

**Additional file 8.** Calculating differences of each methylation sites among 7 clusters

**Additional file 9.** 41 cluster-specific methylation sites and specific methylation sites corresponding to each subtype

**Additional file 10.** Genome annotations of the 41 cluster-specific methylation sites

**Additional file 11.** Functional enrichment analysis and the enriched 30 pathways

## Data Availability

The data sets used and/or analyzed during the current study are available from the corresponding author on reasonable request.

## Abbreviations

LUSC: Lung squamous cell carcinoma
TCGA: The Cancer Genome Atlas
UCSC: The University of California Santa Cruz
NSCLC: Non-small cell lung cancer
TSG: Tumor suppressor genes
CDF: Cumulative Distribution Function
GO: Gene Ontology
KEGG: Kyoto Encyclopedia of Genes and Genomes
AUC: Area under curve
mRNA: messenger RNA
miRNA: microRNA
IPMNs: Intraductal papillary mucinous tumors
